# Immune system gene polymorphisms associated with severe dengue in Latin America: a systematic review

**DOI:** 10.1590/S1678-9946202365058

**Published:** 2023-12-01

**Authors:** Jorge Emilio Salazar Flórez, Ángela María Segura Cardona, Berta Nelly Restrepo Jaramillo, Margarita Arboleda Naranjo, Luz Stella Giraldo Cardona, Ángela Patricia Echeverri Rendón

**Affiliations:** 1 Universidad CES, Grupo de Epidemiología y Bioestadística, Medellín, Colombia; 2 Fundación Universitaria San Martín, Grupo GEINCRO, Sabaneta, Colombia; 3 Universidad CES, Escuela de Graduados, Medellín, Colombia; 4 Universidad CES, Instituto Colombiano de Medicina Tropical, Sabaneta, Colombia

**Keywords:** Dengue, Polymorphism, Immunity

## Abstract

One of the main challenges in the clinical management of dengue is the early identification of cases that could progress to severe forms of the disease. A biomarker that may enable this identification is the presence of genetic polymorphisms in genes associated with immune responses. The objective of this study was to perform a systematic review of the Latin American literature on these genes. An electronic literature search was carried out in PubMed, Scopus, Lilacs, and the Virtual Health Library, and reference lists of systematic reviews in the area. Case-control studies conducted in Latin American countries examining at least one form of genetic polymorphism related to immune responses against severe dengue were included. In total, 424 articles were identified and 26 were included in this systematic review. Of the 26 selected articles, 16 reported polymorphisms associated with the risk of developing severe dengue (Risk); Similarly, 16 articles reported polymorphisms associated with a decreased risk of severe dengue (Protective). The final analysis revealed that multiple polymorphisms in immune system genes were early markers of the progression of dengue in Latin Americans and found that polymorphisms of the TNF-alpha gene may have a critical role in dengue pathogenesis.

## BACKGROUND

The bite of *Aedes* mosquitoes, which can be found in more than 100 countries, transmits an RNA-type virus that causes acute dengue and belongs to the *Flaviviridae* family^1^. It is estimated that about 3 billion people live in areas with increased dengue risk^2^. While around 390 million new cases are reported yearly, roughly 75% are asymptomatic and not included in each nation’s official statistics^3^. This means that about 96 million dengue infection cases occur annually, with symptoms requiring health care attention^4^. In the Americas, 1,173,674 dengue cases were reported in 2021, of which 2,821 (0.24%) were severe dengue cases^5^.

The following factors have been found to increase the severity of the clinical manifestations of dengue: Antibody Dependent Enhancement (ADE)^6^; the immune response mediated by the dengue virus (DENV) serotype that first infects a patient (DENV1, DENV2, DENV3, DENV4) and the order of subsequent infections^7-10^; age at the moment of the disease^11-14^; pre-existing co-morbidities (especially diabetes and renal disease); and the presence of warning signs. Additionally, the clinical signs of a secondary dengue infection are often minor when it occurs less than two years after the first^15,16^; however, intervals between two infections greater than four years have been linked to more severe clinical manifestations^17^.

The presence of allelic variants in the coding sequences for the major histocompatibility complex type B (MIC-B) and for phosphoinositide phospholipase C epsilon 1 (PLCE1)^18^, as well as African ancestry^19,20^, are findings that link the individual’s genetic profile with the severity of dengue in individuals. Other genes control the inflammatory response, such as CD209, which produces the DC-SIGN dendritic cell receptor, and the tumor necrosis factor-alpha (TNF-alpha), a pro-inflammatory cytokine involved in the regulation of immune responses, cell proliferation, differentiation, and apoptosis. These genes also seem to have an essential role in controlling individuals’ susceptibility to severe dengue. Additional examples of genes involved in the control of dengue are the FcRIIA, which codes for Fc-type receptors expressed in antigen-presenting cells and is directly involved in the mechanisms of antibody-dependent enhancement (ADE)^21^, and genes that encode for toll-like receptors (TLRs), which are involved in the activation of innate immunity cells.

No specific treatment for dengue has been established to date, and the development of prophylactic vaccines is still incipient in managing the disease^22^. In fact, incidences of dengue continue to increase, and the virus that causes it is still widely spread^23^. In this scenario, it is essential to continue exploring mechanisms that allow the early identification of severe cases, to improve clinical approaches and directly reduce the mortality rates of the disease. Therefore, we conducted a systematic review of the current literature to identify the genetic variants linked to the emergence of severe dengue (dengue hemorrhagic fever and dengue shock) in Latin American populations.

## MATERIALS AND METHODS

This systematic review was conducted according to the Preferred Reporting Items for Systematic Reviews and Meta-Analyses (PRISMA) guidelines^24^ (Supplementary Table S1). The protocol was not registered before this review.

### Search strategy

An electronic literature search was conducted in PubMed, Scopus, Lilacs, and the Virtual Health Library (VHL - BVS in Spanish). VHL is a specific database for the Americas. A search of the reference lists of systematic reviews in the area was also conducted^25-28^, combining MeSH and DeCS descriptors on dengue, polymorphism and the countries of Latin America. Specific terms were used to search the four databases, emphasizing the search for titles and abstracts. The supplementary material contains the strategy applied to search each database (Supplementary Table S2). English and Spanish terms were combined:

1. Dengue virus OR Dengue OR Severe Dengue
2. Polymorphism, Genetic
3. Argentina OR Argentinian OR Bolivia OR Bolivian OR Brazil OR Brazilian OR Chile OR Chilean OR Colombia OR Colombian OR Ecuador OR Ecuadorian OR Paraguay OR Paraguayan OR Uruguay OR Uruguayan OR Venezuela OR Venezuelan OR Dominican Republic OR Dominican OR Costa Rica OR Costa Rican OR Cuba OR Cuban OR El Salvador OR Salvadorian OR Guatemala OR Guatemalan OR Haiti OR Haitian OR Honduras OR Honduran OR Mexico OR Mexican OR Nicaragua OR Nicaraguan OR Panama OR Panamanian OR Peru OR Peruvian OR Latin America OR Central America OR Caribbean
4. (#1) AND (#2) AND (#3)

### Inclusion and exclusion criteria

Case-control studies conducted in a Latin American country that examined at least one genetic polymorphism related to immune responses to severe dengue were included. Our review only included studies that evaluated polymorphism with well-documented genetic tests: polymorphism detection with PCR-SSP (polymerase chain reaction-sequence specific of primers); RFLP (Restriction Fragment Length Polymorphism) for the restriction enzyme; qPCR (real-time PCR), and the Amplification-refractory mutation system (ARMS-PCR). Articles published up to November 3^rd^, 2022, were included in the review. Studies on non-human genetics (e.g., viral genetics, mosquito genetics), case reports, letters to the editor, and other non-observational studies were excluded. Studies that did not employ confirmatory tests for dengue were also excluded. Tests that adhered to the World Health Organization (WHO) guidelines^29,30^, using methods such as viral isolation techniques, detection of antigens or antibodies, and nucleic acid detection, were considered appropriate. Lastly, studies using laboratory-confirmed IgM ELISA or IgG ELISA and reverse transcription polymerase chain reaction (RT–PCR) methods were also included.

### Study selection

Two authors (JESF and LSGC) independently reviewed titles and abstracts and identified potentially relevant articles, resolving discrepancies through further review and mutual consensus. Both investigators fully read all potentially relevant articles and determined the final reports to be included in this review. The data sets were extracted and organized in bibliographic tables. The primary fields contained information on the authors, country, year of publication, sample size for each study group, study objective, molecular test to determine the genetic polymorphism, the polymorphism evaluated, the group and type of immunity and WHO classification of the dengue cases (guideline 1997 or 2009). Types of immunity were classified according to Immunity Groups, which were based on the contributions of Harapan *et al*.^3^, and Bhat *et al*.^31^: Group A) Innate immunity; Group A1) Interferons and interleukins; Group A2) Mannose-binding lectin (MLB2); Group A3) Others (histidine, serotonin, complement and nitric oxide); and Group B) Genetic (HLA).

### Quality assessment

The quality of each study was assessed using the Newcastle-Ottawa Quality Assessment Scale for Case-Control Studies (NOS)^32^, which evaluates the selection, comparability, and exposure determination of each study, and independently assessed by two authors (JESF and LSGC). Based on the NOS score and quality classification, the risk of bias in the initially selected studies was evaluated and classified as high risk (NOS≤6), some concerns (NOS=7), and low risk (NOS≥8). The study quality evaluation was summarized in a figure specifying whether studies met the criteria (green: low risk of bias), did not meet the criteria (red: high risk of bias), or if results were unclear (yellow: unclear risk/some concerns/lack). Supplementary Table S3 shows the scoring criteria based on the Newcastle scale.

## RESULTS

### Search results and article selection

**Figure 1:**
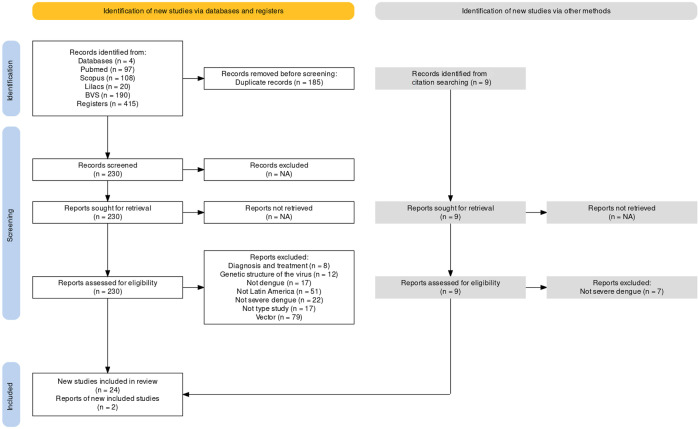
PRISMA flowchart of the strategy used to identify papers assessing polymorphism genetics and severe dengue

In total, 424 bibliographic references were found: 97 in PubMed, 108 in Scopus, 20 in Lilacs, and 190 in VHL. Nine articles were obtained during the review of reference lists in systematic reviews of the area. A total of 185 studies were discarded due to duplication. Then, the remaining 230 articles were thoroughly reviewed. As a result, 26 articles met all the inclusion criteria (Figure 1).

### Study characteristics

In total, 12 (46.2%) of the selected studies were conducted in Brazil^33-44^, seven in a Mexican population^45-51^, five in Cuba^19, 52-55^, and two with a Venezuelan population^56,57^. All of them were case-control studies, but one nested in a cohort^41^. Most studies applied WHO criteria to classify dengue cases^29,30^, with only two articles not reporting this data^44,57^. Fifteen articles used the 1997 WHO dengue classification criteria^19,33-35,38,43,47-53,55,56^, while only six considered the 2009 guidelines^36,39-42,46^. The remaining three studies applied the WHO guidelines from 1994^54^, 2004^37^, and 2008^45^ (Table 1).

**Table 1:** Characteristics of the included papers assessing polymorphism genetics and severe dengue.

**Article**	**Country, year**	**Objective**	**Population**	**Dengue classification system**	**Molecular testing for polymorphism**	**Immunity group**	**Immunity type**	**Polymorphism assessed**	**Protective or risk polymorphisms**
Silva *et al*.^33^	Brazil, 2020	To evaluate the associations between the IFNL3 gene (rs12979860) and the clinical outcomes in children with dengue.	DF: 96 DHF/DFC: 110	WHO, 1997	qRT-PCR using TaqMan	A1	Innate immunity (interferon lambda 3)	rs12979860	Inconclusive: rs12979860. Risk: allele T of IFNL3 gene
Santos *et al*.^34^	Brazil, 2019	To assess whether SNPs in serotonin and nitric oxide were associated with the risk of progression of dengue hemorrhagic fever in a sample of Brazilian patients.	DF: 78 DHF: 49	WHO, 1997	qRT-PCR using TaqMan	A3	Innate immunity (nitric oxide and serotonin)	rs1799983	Protective: rs1799983, rs2430561 y rs25531
Falcón *et al*.^45^	Mexico, 2009	To analyze possible associations between HLA class I and II polymorphisms and the severity of the clinical disease caused by dengue virus infection in mestizo Mexican patients.	DF: 23 DHF: 16 Controls: 34	WHO, 2008	PCR-SSO and nucleotide sequencing method for subtype identification	B	Genetic	HLA	Risk: HLA-B and HLA-DQB1
Figueiredo *et al*.^35^	Brazil, 2016	To evaluate the influence of MBL2 polymorphisms as a modifier condition of dengue progression	DF: 104 DHF:57	WHO, 1997	qRT-PCR using TaqMan	A2	Innate immunity (MBL2)	MBL2 polymorphisms	Risk: OO genotype and O allele (low production of MBL2)
García *et al*.^52^	Cuba, 2010	To investigate the role of the FcγRIIa polymorphism in a group of Cuban individuals with a history of DH, FD, or asymptomatic dengue.	DF:68 DHF/DSS:29 Subclinical: 42	WHO, 1997	PCR-SSO	B	Genetic	FcγRIIa polymorphisms	Protective: genotype RR131 Risk: genotype HH131
Noecker *et al*.^46^	Mexico, 2014	To characterize the frequency and distribution of the FcγRIIa and DC-SIGN polymorphisms and to compare their distribution among asymptomatic, uncomplicated, and severe dengue cases in Morelos, Mexico.	Asymptomatic: 145 uncomplicated: 67 Severe dengue:36	WHO, 2009	qRT-PCR using TaqMan	A3	Innate immunity (histidine)	rs1801274, rs4804803	Protective: rs1801274 Risk: rs4804803
Ornelas *et al*.^36^	Brazil, 2019	To investigate the association between the MBL2, CLEC5A, ITGB3 and CCR5 genes and the severity of dengue in children	Dengue severe cases: 87 Controls:197	Brazilian Ministry of Health. WHO, 2009	qRT-PCR using QuantStudio	A2	Innate immunity (MBL2)	rs7095891, rs1800450, rs1800451, rs4935047, rs930509, rs2120131 and rs2099902	Risk: rs7095891G, rs1800450C, rs1800451C, rs4935047A, rs930509G, rs2120131G, rs2099902C, rs4935047G and rs7095891G
Pastor *et al*.^37^	Brazil, 2013	To determine the relationship between allele/haplotype variants of the CFH gene and the clinical outcome in patients with DENV-3 dengue infection	DF:34 DHF:87 Healthy: 93	WHO, 2004	PCR amplification reaction using GeneAmp Genotyping the C-257T PCR using TaqMan	A3	Innate immunity (complement factor H)	rs800292, exon 14 rs3753396 exon 19 and rs1065489	Protective: rs3753394 and rs800292
Santos *et al*.^38^	Brazil, 2017	To evaluate the influence of IL-10, TNFA and IFNG gene polymorphisms on the susceptibility to dengue infection or progression in a sample of Brazilian patients	DF: 78 DHF: 49 Healthy controls: 135	WHO, 1997	qRT-PCR using TaqMan; and amplification refractory mutation system-PCR	A1	Innate immunity (TNFA, IL10 e INFG)	rs3753394 (C-257T), rs800292 (G257A), rs3753396 (A2089G) and rs1065489 (G2881T)	Protective: rs2430561 Risk: rs180871
Vargas-Castillo *et al*.^47^	Mexico, 2018	To identify the association between seven gene polymorphisms related to the immune response and severe presentations of dengue infection in patients from an endemic region of Mexico	DF: 138 DHF: 31 Healthy controls: 304	WHO, 1997	qRT-PCR using TaqMan	A1	Innate immunity (TNF)	rs1800629 (TNF), rs4804803 (CD209), rs2780831 (JAK1), rs1801274 (FCGR2A), rs231775 (CTLA4), rs12979860, and rs8099917	Risk: rs1800629
Xavier-Carvalho *et al.* ^39^	Brazil, 2013	To evaluate the impact of TNF, IL-10, MIF, DC-SIGN, CLEC5A, NOD2, CCR5 and MRC1 polymorphisms on patients’ susceptibility to dengue infection and on the progression of this disease	SD: 88 Healthy controls: 335	WHO, 2009	qRT-PCR using TaqMan	A1	Innate immunity (TNF, CLEC5A)	rs1800629, rs1800871, rs4804803, rs755622 rs333, rs1926736 rs2066843 and rs751271	Protective: rs4804803 Risk: rs1285933
Xavier-Carvalho *et al*.^40^	Brazil, 2017	To determine the association between the CLEC5A polymorphism and severe dengue infection in a Brazilian population, in order to investigate the functional effect of CLEC5A in an in vitro experiment with blood samples from patients infected with dengue	SD: 151 Mild dengue: 62	WHO, 2009	qRT-PCR using TaqMan	A3	Innate immunity (CLEC5A)	rs1285933	Risk: rs1285933
Azevedo *et al*.^41^	Brazil, 2019	To verify the relationship between the single nucleotide polymorphism (SNP) of the G2431A IDO1 gene (rs3739319) and the development of severe dengue.	395 DWOS:131 DWS:143 SD:25 Healthy: 96	WHO, 2009	qRT-PCR using TaqMan	B	Genetic (AA IDO1)	rs3739319	Protective: rs3739319GG Risk: rs3739319AA
Oliveira *et al.* ^42^	Brazil, 2014	To determine the possible association between the rs4804803 polymorphism and the dengue virus and its pathogenesis.	FD: 156 DHF: 12 Controls: 72	Brazilian Ministry of Health. WHO, 2009	qRT-PCR using TaqMan	B	Genetic	rs4804803	Protective: rs4804803GG
Silva *et al*.^43^	Brazil, 2010	To identify the genes associated with the clinical presentation of dengue.	DHF: 50 FD:236 Asymptomatic :236	Brazilian Ministry of Health. WHO, 1997	BeadArray technology	A1	Innate immunity (INF1)	rs11208534, rs2780831 and rs310196	Protective: rs11208534, rs2780831 and rs310196
Fernández-Mestre *et al*.^56^	Venezuela, 2009	To analyze the frequency of HLA class I (-A, -B and -C) and class II (-DRB1) polymorphisms in Venezuelan patients with FD and DHF and the relationship between these polymorphisms and the clinical manifestations of the disease.	DF: 43 DHF: 28 Not clinically classified:6 Healthy controls:127	WHO, 1997	PCR-SSO reverse using the Dynal RELI	B	Genetic	B*15, B*49, DRB1*02 and DRB1*03	Protective: A*03 Risk: B*57 and B*40
Posadas-Mondragón *et al.* ^48^	Mexico, 2020	To explore the association between SNPs in TLRs and the clinical forms of dengue in the Mexican adult population	DF:100 DHF:65 Healthy adults:89	WHO, 1997	qRT-PCR using Applied Biosystems genotyping assays.	A3	Innate immunity (Receptor Toll)	rs3775291, rs4986791, rs4986790, rs3764880, rs6552950, rs2737190, rs11536865, rs179008, rs3853839, rs5741883, rs1548731 and rs10983755	Protective: TLR4-rs2737190-G/G and TLR4-rs11536865-G/C
Sánchez-Leyva *et al*.^49^	Mexico, 2017	To evaluate the relationship between the polymorphisms of genes -308 and -238 of the tumor necrosis factor alpha (TNF-α) and its circulating serum levels, and patients’ susceptibility to dengue virus infection and its different clinical and laboratory manifestations in an endemic region of Mexico.	DF: 182 DHF: 69 Controls: 275	WHO, 1997	PCR - RFLP	A1	Innate immunity (TNF-α)	TNF-308 and TNF-238	Protective: -308G/A
García *et al.* ^53^	Cuba, 2011	To analyze the polymorphisms of the non-classical HLA class I MICA-MICB genes in Cuban adults infected with DV-4 during the 2006 epidemic.	DF:68 DHF:36 Asymptomatic:42 Control population:155	WHO, 1997	PCR amplification using Taq	B	Genetic	MICA and MICB	Risk: MICA*008 and MICB*008
García-Trejo *et al*.^50^	Mexico, 2011	To evaluate the relationship between polymorphisms of the TNF-α gene and genetic susceptibility to dengue in a group of mestizo patients from the State of Morelos, Mexico.	DF:85 DHF:45 Healthy controls:169	WHO, 1997	PCR - RFLP	A1	Innate immunity (TNF-α)	TNF-308 and TNF-238	Protective: TNFA - 238A
Sierra *et al*.^54^	Cuba, 2007	To examine the HLA-A/B class I and HLA-DRB1 class II polymorphisms in Cuban individuals with a history of DF or DHF during primary and secondary dengue 2 infections during the 1997 outbreak.	DF:73 DHF:47 Healthy controls: 189	WHO, 1994	PCR-SSP	B	Genetic	HLA-A/B class I, and HLA-DRB1 class II	Protective: HLA-DRB1 Risk: HLA-I
Pérez *et al*.^55^	Cuba, 2010	To analyze the polymorphisms of cytokine genes in a group of individuals who developed DHF during the 1997 epidemic.	DHF: 43 Healthy controls: 99	WHO, 1997	PCR-SSP	A1	Innate immunity (TNF-α)	TNF (308 A/G), IFN (874A/T), TGF-1 (codon 10 T/C and codon 25 G/C), IL-10 (1082 A/G, 819 C/T, 592 A/C), IL-6, 174 G C)	Risk: TNF- (308) GG and TGF-1 (c25) GG
Sierra *et al*.^19^	Cuba, 2017	To demonstrate whether the expression of OSBPL10 protects individuals against dengue infection in a Cuban population of African descent.	Habana: DF:36 DHF:31 Asymptomatic: 32 Healthy: 47 Guantanamo: DF:41 DHF:29 healthy: 42 Asymptomatic: 16	WHO, 1997	qRT-PCR using LightCycler RNA	B	Genetic	RXRA y OSBPL10	Protective: LXR/RXR
Santos *et al*.^44^	Brazil, 2020	To identify the effect of the SNPs TNF-α-308G/A and -238G/A on a population from north-eastern Brazil.	DF:108 DHF:50 Controls: 123	Not reported	qRT-PCR using TaqMan; and Fast-Time PCR Thermocycler	A1	Innate immunity (TNF-α)	TNF -308G/A and -238G/A	Protective: SNP-308G/A and SNP238A/A Risk: SNP-308G/G and SNP238G/A
LaFleur *et al*.^51^	Mexico, 2002	To determine the association between HLA-DRB1 alleles and dengue hemorrhagic fever in Mexico.	DHF:34 DF:47	WHO, 1997	PCR-SSO reverse	B	Genetic	HLA-DRB1 HLA-DR4	Protective: HLA-DR4
Fernadez-Mestre *et al*.^57^	Venezuela, 2004	To analyze selected single-nucleotide polymorphisms (SNPs) of several cytokine genes [(TNF)-a, (IFN)-g, (IL)-6, (TGF)-b1 and IL-10)] in patients with dengue virus infections and to assess their relationship with patients’ susceptibility to dengue virus disease.	DHF:25 DF:41	Not reported	PCR-SSP	A1	Innate immunity (TNF-α)	TNF-308A, INFG, IL10 e IL6	Risk: TNF-308A

Immunity groups = Group A: Innate immunity, Group A1: Interferons and interleukins, Group A2: Mannose-binding lectin (MLB2), Group A3: Others (histidine, serotonin, complement and nitric oxide), Group B: Genetic (HLA); DF = dengue fever; SD = severe dengue ; DHF = dengue hemorrhagic fever; DFC = DF complicated; DSS = dengue shock syndrome; DWOS = dengue without warning signs; DWS = dengue with warning signs; qRT-PCR: real-time polymerase chain reaction; PCR-SSP: polymerase chain reaction-sequence specific primer; PCR-SSO = polymerase chain reaction–sequence-specific oligonucleotide; PCR = polymerase chain reaction; RFLP: restriction fragment length polymorphism.

The number of people diagnosed with severe dengue in different studies ranged from 16^45^ to 143^41^. Also, studies frequently included the general population and asymptomatic cases in their analyses^19,36-39,41-50,52-56^. The article by LaFleur *et al.* ^51^ is the oldest study in our review: it was carried out in 2002, while the most updated studies were conducted in 2020^33,44,48^ (Table 1).

**Figure 2:**
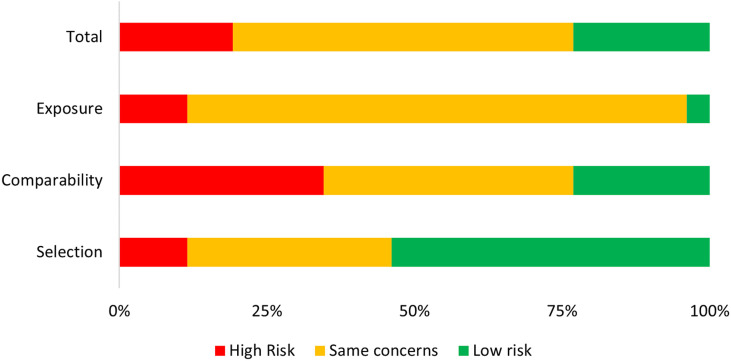
Summary of the assessment of risks of bias in the included articles. The classification is based on the Newcastle-Ottawa Quality Assessment Scale for Case-Control Studies (NOS).

The articles included in this review analyzed polymorphisms based on different immunological classifications: ten articles assessed polymorphisms with type A1 immunity (interferons and interleukins)^33,38,39,43,44,47,49,50,55,57^; A2 immunity (Mannose-binding lectin - MLB2) was evaluated by Figueiredo *et al.* ^35^, Ornelas *et al.* ^36^, and LaFleur *et al.* ^51^; and eight articles included analyses on immunity group B (HLA Genetics)^19,41,42,45,52-54,56^. The remaining articles covered other immunities, including histidine, serotonin, complement, and nitric oxide^34,37,39,46,48^ (Table 1).

Eight of the studies in our systematic review specifically investigated the association between TNF-alpha gene polymorphisms and severe dengue in Latin America. These studies focused on various single nucleotide polymorphisms (SNPs) within the TNF-alpha gene, including -308G>A, -238G>A, -857C>T, and -1031T>C. The results of these studies varied: while some reported significant associations between specific TNF-alpha SNPs and severe dengue, others found no significant correlations between these two factors. This heterogeneity may have resulted from differences in study populations, sample sizes, and genotyping techniques.

Table 1 shows that the most used genotyping method was the real-time polymerase chain reaction (qRT-PCR), employed in 14 studies^19,33-36,38-42,44,46-48^. Four studies used the polymerase chain reaction–sequence-specific oligonucleotide (PCR-SSO)^45,51,52,56^. Other genotyping methods were the polymerase chain reaction with sequence specific primers (PCR-SSP)^54,55,57^ and the polymerase chain reaction-restriction fragment length polymorphism (PCR-RFLP)^49,50^.

### Quality of the studies

In total, 46.2% of the 26 articles (n=12) presented some concern or risk of bias in their selection of cases and controls. The selection of cases was mainly based on WHO criteria from 1997 or 2009, with clinical confirmation through RT-PCR tests. In their assessment of comparability between groups, 34.6% of studies had a high risk of bias. Lastly, 96.2% of the studies presented some concern or a high risk of bias in their exposure assessment—they did not present any information on losing participants. Supplementary Table S4 presents the eight quality review criteria for each study included in this review.

## DISCUSSION

This systematic review demonstrated the existence of multiple polymorphisms in immune system genes that are related to the clinical outcomes of dengue virus infections. As Table 1 summarizes, 17 of the 26 articles reported finding polymorphisms that are associated with the risk of severe dengue (Risk) and 17 articles reported detecting polymorphisms that are associated with a decreased risk of severe dengue (Protective). Notably, most of these studies were conducted in Brazil (n=12).

Regarding the immune response in dengue virus infection, both innate and adaptive responses play an essential role in defending organisms infected with severe dengue, and that the regulation of these responses directly impacts the clinical outcome of the disease^58^. One of the first mechanisms employed by the innate immune system is the production of interferon and proinflammatory cytokines to trigger the initial response against the virus through dendritic cells^59^. However, this mechanism may lead to cell permeability and fluid leakage.

Most genes reported in the analyzed studies are related to the innate immune response. Polymorphisms in Toll-like receptors responsible for recognizing viral proteins^48^; genes associated with the production of interleukins capable of inhibiting the synthesis of proinflammatory cytokines and suppressing the ability of cells to present antigens^33,34,38,39,43,44,49,50,55,57^; and genes coding for surface proteins in multiple cells of the immune system, such as the type C lecithin receptor (DC-SIGN)^39,46,47^, MBL2^35,36^ (it should be noted that Ornelas *et al.* ^36^ only observed such an association after haplotype analyses), and CLEC5A^39,40,46,47^. One study also reported a protective effect mediated by complement^37^.

The substantial number of studies focused on TNF-alpha gene polymorphisms in this review highlights the importance of this cytokine in severe dengue pathogeneses. The inconsistent findings among these studies call attention to the complexity of the role of TNF-alpha in individuals’ susceptibility to severe dengue and the need for further investigation. Larger, multi-center studies with standardized methodologies and thorough genetic analysis are needed to clarify the association between TNF-alpha gene polymorphisms and severe dengue risks. Future research should also explore the role of interactions between TNF-alpha polymorphisms and other immune system genes in the pathogenesis of dengue, to further elucidate the genetic factors influencing individuals’ susceptibility to severe dengue.

Concerning cellular immunity, the activation of CD4+ and CD8+ T lymphocytes is essential for eliminating infected cells. However, T cells may cause immunopathology during DENV infections, in a phenomenon called original antigenic sin, in which the activation of memory lymphocytes generates an elevated production of proinflammatory cytokines with the consequences described above^60^. Studies by Falcón *et al.* ^45^, Fernández-Mestre *et al.* ^56^, García *et al.* ^53^, and Sierra *et al.* ^54^ documented the influence of polymorphisms in the major histocompatibility complex responsible for the regulation of the immune system through the process of antigen presentation. García *et al.* ^52^ in 2010 and Noecker *et al.* ^46^ in 2014 also investigated this immune system and identified polymorphisms in the FcγRIIa gene, which encodes cell surface proteins that mediate responses in B lymphocytes, follicular dendritic cells, natural killer cells, macrophages, neutrophils, eosinophils, basophils, among others. Other studies in our review focused on verifying the relationship between genetic immunity and the single nucleotide polymorphism (SNP) of G2431A IDO1, OSBPL10, and HLA-DRB1^19,41,42,51^.

A previous systematic review demonstrated that genetic variations within MICB (meta-OR=2.35, 95% CI: 1.68–3.29), MBL2 (meta-OR=1.54, 95% CI: 1.02–2.31), and IFN-γ (meta-OR=2.48, 95% CI: 1.30–4.71) are associated with dengue^25^; however, it did not differentiate between severe dengue and classic dengue or asymptomatic dengue. Another review analyzed different associations between DC-SIGN Promoter-336G/A (rs4804803)^25^ and concluded that dendritic cell-specific intercellular adhesion molecule-3-grabbing non-integrin (DC-SIGN) promoter-336G/A (rs4804803) polymorphism is associated with severe dengue. A study included in our review reported that rs4804803 behaved as a risk factor in Mexican people^46^, while as protective in Brazilian people^39,42^.

We noted some similarities and differences between the studies we analyzed and other studies concerning innate immunity polymorphisms: some reports from Thailand indicated that rs4804803 (CD209) and rs3753394 (CFH) polymorphisms were not linked to dengue^25^. Similarly, the current review found no significant association between this polymorphism and severe disease in Latin American countries^38,47^. However, another study conducted with the Thai population confirmed CD209 with an OR=5.84 (2.77–12.31) of DHF compared to DF^25^.

While analyzing this same component of innate immunity, a study conducted in India confirmed OR=0.39 (0.16-0.88) of severe disease associated with rs3775291 (TLR3)^25^. In contrast, no association between these factors was found in the Latin American population^48^. Lastly, contrary to what was found in Indonesia regarding the TLR4 gene (no significant association with disease)^25^, our study confirmed a protective effect of TLR4- rs2737190- G/G/G in cases of severe dengue fever^48^.

While analyzing the genetic immunity groups, our study found significant associations of the MICB gene with the risk of severe dengue^53^. Previous studies conducted with the Asian population observed a similar scenario, confirming that the same increase in risk affected DSS: 1.58 (1.02–2.40) odds of DSS compared to non-DSS^25^. Differences between countries in Latin America and those in other continents may occur due to specific ethnicity factors resulting in cases in which Asians are protected by polymorphisms but Latin Americans are not, or on the contrary, for some polymorphisms the protective effect occurs in Latin Americans but not in Asians.

Dengue is currently a public health problem in most Latin American countries. Since 2009, the disease has expanded its distribution, causing periodic epidemics with a constant raise in cases. Identifying the polymorphisms that affect dengue can help researchers find early markers that make it easier to predict the clinical outcome of this disease and may even be helpful in designing vaccines. Genetic studies are essential for gathering information on circulating viruses and creating a better understanding of DENV transmission and epidemiology in a specific region.

Multiple limitations influence the comparison of studies in this review: an example is the low representativeness of the samples in most studies. In effect, only 11 studies reported the power calculations they used to estimate SNP differences between cases and controls or presented the limitation of small samples^33,34,36,38,39,41,44-48^. Thus, few studies conducted multiple testing or applied methods of correction for small samples, such as Bonferroni’s correction, Welch’s correction, or Yates’ correction^19,37,41-43,53,55-57^.

Other limitations of our review include the variability in the classification of severe disease used in the studies we evaluated, which ranged from the 1994 WHO guidelines^54^ to the more updated 2009 classification^36,39-42,46^, and the fact that certain studies not specified which standard they used^44,57^. Additionally, the differences in the methods used to classify polymorphisms resulted in uncertainty in our analysis. Although more than half of the studies used the real-time polymerase chain reaction (qRT-PCR)^19,33-36,38-42,44,46-48^, others also applied the PCR-SSO, PCR-SSP, or PCR-RFLP.

## CONCLUSION

Lastly, it is crucial to consider the time span of the studies included in this review, where the most recent studies occurred in 2020^33,44,48^, and some were conducted almost two decades ago. The different biases previously described for each of the studies resulted in a high subjective heterogeneity. On the other hand, the lack of studies in most Latin American countries, probably due to low research funding, has hampered a thorough investigation of the potential of markers in preventing severe dengue in this region.

Nevertheless, this review provides an overview of the genetic aspects associated with severe dengue in this region, which is an essential analysis, considering the diversity that could be expected in this regard and the fact that these types of studies are mainly published in Asian countries. Despite the aforementioned scenario, the efforts made to understand the clinical course of patients with dengue are fully justified. The evidence collected so far will hopefully serve as a basis for improving disease prediction methods, positively affecting the early identification of cases that require greater health care attention.
